# A torque induced iatrogenic fracture of the humeral shaft in proximal humeral fracture plating – A case report and biomechanical perspectives

**DOI:** 10.1016/j.amsu.2021.102615

**Published:** 2021-07-27

**Authors:** Ali Lari, Mohammad Alherz, Ali Jarragh

**Affiliations:** a AlRazi Orthopedic Hospital, AlSabah Medical Region, Kuwait; b Farwaniya Hospital, Farwaniya Governorate, Kuwait; c Department of Orthopedic Surgery, Jaber Alahmed Alsabah Hospital, South Surra, Kuwait

**Keywords:** Proximal humerus, Iatrogenic fracture, Complication, Locking plate, Torque

## Abstract

**Introduction:**

Proximal humerus fractures are common amongst the elderly and osteoporotic cohorts. Common treatment methods include proximal locking plates. In this case, we describe an iatrogenic fracture of the proximal humeral shaft during screw insertion under power. Similar cases have not been described previously in open reduction and internal fixation of a proximal humerus fracture. Further, we focus particularly on precautionary measures that aim to avoid such complications that may lead to considerable morbidity.

**Case presentation:**

We describe a case of a 65 year old osteoporotic female who underwent open reduction internal fixation of a proximal humerus fracture complicated by an unusual iatrogenic humeral fracture at the level of insertion of the distal screw, likely secondary to inserting the proximal locking screws under power.

**Conclusion:**

In this case, we explore the possible factors leading to the fracture and precautionary measures to avoid them. The rate of iatrogenic intraoperative fractures are likely underreported and have not been described in open reduction and internal fixation of an existing fracture. The underlying factors that may predispose to such complications have not been previously described in similar cases. This case serves as a warning of an unanticipated complication and describes the potential biomechanical factors involved.

## Highlights

• Proximal humerus fractures are common in the elderly and osteoporotic cohorts.
• Iatrogenic fractures intraoperatively during open reduction internal fixation have not been reported in orthopedic trauma.
• Care should be taken to avoid excessive energy/torque at screw sites while using electric drivers.
• The biomechanical precautions during fixation should be visited and more extensively described to avoid similar incidents.

## 1 Introduction

Proximal humerus fractures are common amongst the elderly and those with osteoporosis [1]. Depending on fracture severity, age, osteoporotic status and surgeon's preference, locking plates present one of the management options, none of which are currently favoured by a sufficient evidence base [2]. The likely underreported iatrogenic complications of locking plates include articular screw penetration, varus collapse and sub-acromial impingement [3,4]. We describe a proximal humerus fracture complicated by a shaft fracture secondary to the insertion of locking screws using an electric driver. To our knowledge, similar cases have never been reported before outside the scope of arthroplasty. Illustrations were created to clearly demonstrate the fracture method. This case report has been submitted in line with the SCARE criteria [6].

## 2 Case report

**Fig. 1:**
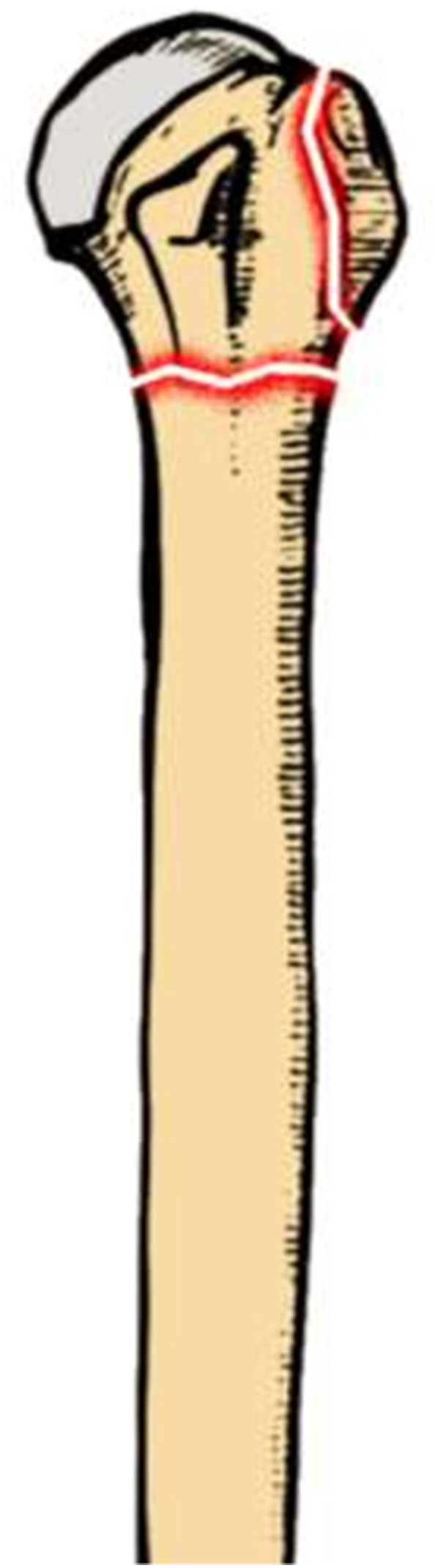
Illustration displaying the proximal humeral fracture pattern before reduction.

A 65 year old female with a background of hypertension and osteoporosis presented to the orthopedic department complaining of pain and reduced range of motion after slipping and falling directly onto her left shoulder. Initial assessment showed mild bruising and tenderness, while neurovascular status was intact with no open wounds. Subsequent imaging showed a displaced two-part proximal humerus fracture [Fig. 1]. A joint decision was made to undergo open reduction and internal fixation using a proximal humerus locking plate (Philos plate®).

**Fig. 2:**
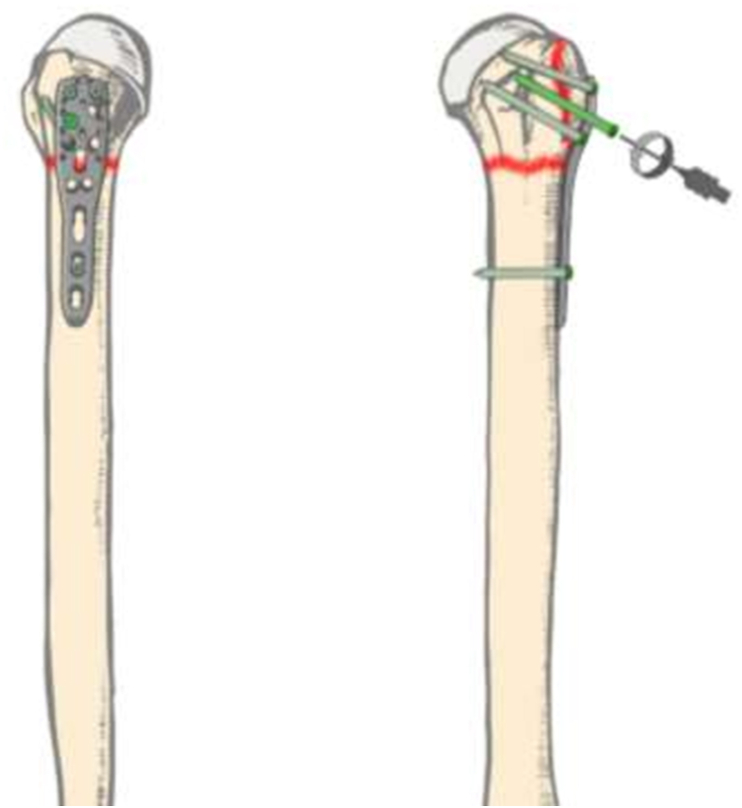
–AP & Lateral illustrations of the proximal humerus plate applied along with three inserted proximal screws and one distal screw. In this image, the fourth proximal locking (bright green) screw is being inserted under power by use of an electric driver (Right). (For interpretation of the references to colour in this figure legend, the reader is referred to the Web version of this article.)

The patient was prepared supine under general anesthesia. The fracture was accessed uneventfully via a delto-pectoral approach. Open reduction was achieved and a short proximal humeral locking plate was fixed preliminarily with K-wires. Next, one cortical screw was inserted distally and three proximal locking screws were inserted under power, with the last few turns performed manually [Fig. 2].

**Fig. 3:**
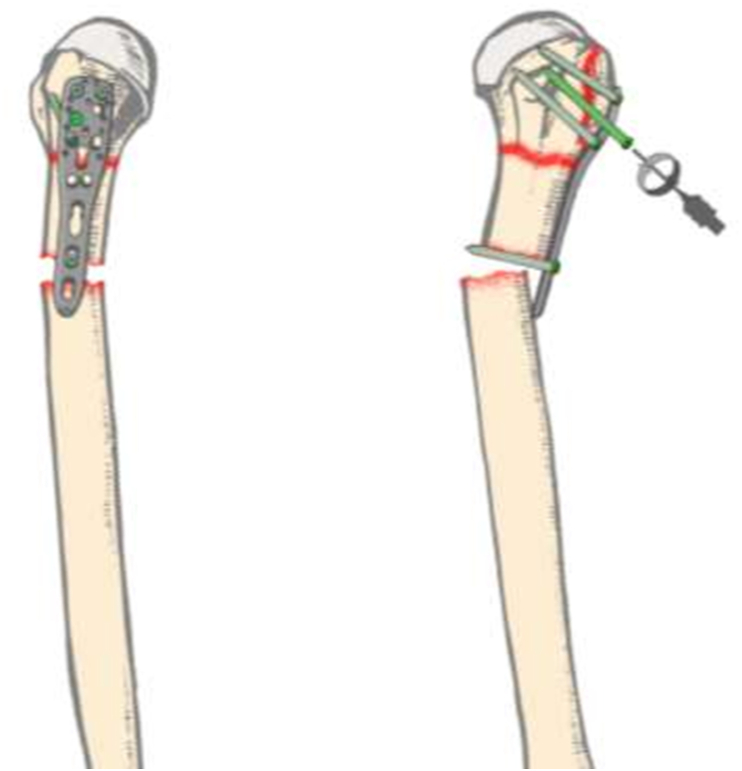
–AP & Lateral illustrations of the subsequent humeral shaft fracture at the level of the distal cortical screw while inserting the fourth proximal locking screw under power.

During insertion of the fourth proximal locking screw under power, maximum screw purchase was obtained prematurely. This resulted in a violent sudden flexion of the humerus with the screw as a pivot, followed by an ominous snap [Fig. 3]. Fluoroscopy confirmed a significantly displaced fracture of the humeral shaft along the entry of the distal cortical screw. The metal was subsequently removed, reduction obtained and a longer proximal locking plate, resulting in a lengthened and more complex surgery.

## 3 Discussion

Inserting long screws under power is common practice and serves to reduce operative duration whilst relieving muscle fatigue. In the above case, we describe an unusual occurrence where the sudden purchase of the screw resulted in rotational forces along the axis of the plate, displacing the humerus anteriorly with subsequent fracture at the level of the cortical screw. Similar cases have not been reported in the literature. Intraoperative iatrogenic fractures appear to be more extensively reported in arthroplasty and hip fractures [5].

Factors which may be implicated in the aetiology of this fracture include the absence or failure of torque limiting devices in drivers, which are often equipped to disengage the rotational movement when the torque requirement for driving the screw is excessive. Secondly, the distal cortical screw may have resulted in an undetected undisplaced fracture in the osteoporotic bone upon insertion. Additional precautionary measures that may have avoided the fracture include stabilizing the arm during insertion, driving the screw at the lowest speeds and insertion of another distal screw to support the construct and reduce stress risers.

The incidence and underlying factors associated with iatrogenic fractures are poorly described and likely underreported, potentially due to the nature of the event being viewed as purely a complication with little focus on the educational aspects. The above case serves as a warning of possible pitfalls during open reduction and internal fixation of osteoporotic bone.

## 4 Clinical message

Intraoperative iatrogenic fractures during ORIF are rare and likely underreported. These complications are usually unanticipated and may result in avoidable morbidity. Precautionary measures should be undertaken and the respective biomechanical basis explore thoroughly, particularly in osteoporotic bone.

## Ethical approval

This study was exempt from ethical approval – observational retrospective case report.

## Funding

No funding was received.

## Author contribution

Ali Lari – Writing, conceptualization, Mohammad Alherz – Writing, review, Ali Jarragh – Conceptualization, review, supervision.

## Consent

Written informed consent was obtained from the patient for publication of this case report and accompanying images. A copy of the written consent is available for review by the Editor-in-Chief of this journal on request.

## Author contribution

Ali Lari – Writing, conceptualization, Mohammad Alherz – Writing, review, Ali Jarragh – Conceptualization, review, supervision.

## Registration of research studies

Exempt from registration.

## Guarantor

Dr Ali Lari.

## Declaration of competing interest

The authors declare no conflict of interest.
